# A double blind randomized controlled trial to evaluate the efficacy of green tea in gingivitis

**DOI:** 10.6026/97320630017805

**Published:** 2021-09-30

**Authors:** Nidhi Thakur, Vishal Kumar, Ankita Agarwal, Soni Kumari

**Affiliations:** 1 Department of Dentistry, Anugrah Narayan Magadh Medical College and Hospital, Gaya, Bihar, India; 2 Department of Orthodontics, Hazaribagh Dental College and Hospital, Hazaribagh, Bihar, India; 3 Department of Dentistry, Nalanda Medical College and Hospital, Patna, Bihar, India

**Keywords:** alternative therapy, gingival index, green tea, orthodontic treatment

## Abstract

Gingivitis is the most common form of oral disease especially among patients undergoing fixed orthodontic treatment. Green tea, which is extensively used in Asian countries, can help to improve the overall gingival health, which can be assessed by using the gingival indices. Evaluation of the effectiveness of green tea on the gingival health of patients undergoing Orthodontic treatment is of interest. 40 otherwise healthy patients undergoing fixed orthodontic treatment were randomly divided in two groups namely (1) study group and (2) control group. Gingival indices were scored for all the patients. Study group was given mouth rinse with green tea extract and control group was given placebo with no green tea extract. Gingival indices were measured for all the patients after 21 days. Mann Whitney U test and Wilcoxon test was used for statistical analysis. The gingival indices scoring in which the values before and after the use of mouthwash were compared. The p value was found to be statistically significant (p<0.05) in study group. But in control group statistical significant could not be reached.

## Background:

Certain plants used in folk medicine serve as a source of therapeutic agent by having multiple effects in addition to their antimicrobial activity. Herbal formulations can provide an option for safe and long-term use. Green tea is one such herbal product, which has long been a popular beverage in Asian culture with origins dating back over 5000 years [1]. Ancient Chinese and Japanese medicine believed green tea consumption could cure diseases and heals wounds and have shown the potential benefits of drinking green tea. Routine consumption of green tea may help promote healthy teeth and gingiva due to its antioxidant properties. Green tea is prepared from unfermented leaves. The more the leaves are fermented, the lower the polyphenol content and the higher the caffeine content. Green tea has the highest polyphenol content. Green tea reportedly contains the highest concentration of powerful antioxidants called polyphenols.

Gingivitis is the most common form of oral disease. A definition in literature states gingivitis is inflammation of gingiva in which junctional epithelium remains attached to tooth at its original level [3]. Gingivitis is caused by the accumulation of plaque on the tooth and gum surfaces [4]. Experimental gingivitis studies have proved the role of plaque in the aetiology of periodontal infections and demonstrated direct relationship between plaque levels and the development of human gingivitis [5,6]. Plaque is a colorless, sticky film containing millions of bacteria and their by-products, which constantly grows on the teeth. Not flossing also contributes to developing gingivitis. Poor oral hygiene practice influences the prevalence of gingivitis [3].

Gingivitis is characterized by inflammation and bleeding of the gums [3,6]. As gingivitis is rarely painful in its early stages, it often goes unnoticed until severe irritation or receding of gums occurs. Gingivitis results in red discoloration of gingival tissues. If left untreated, it may lead to a more serious condition called periodontitis, in which the inner gum and bone pull away from teeth and form pockets [3]. These pockets can collect bacteria and debris, and become infected or abscessed. Bacterial toxins eventually break down the underlying bone and connective tissue that hold teeth in place. The result is swollen, tender gums, which bleed easily when, brushed and can ultimately lead to tooth loss. Gingivitis is measured by gingival index, and one such index is Loe and Silness index [7].

Mechanical plaque control, like scaling and root planing is the recommended step in the management of gingivitis, but there are factors such as accessibility or presence of plaque retentive areas, that can limit microbiological response [8]. Many chemical agents have been tested as adjuncts to mechanical methods, which can reduce plaque and its associated gingivitis. Several antibacterial chemicals like chlorhexidine, showed side effects such as discoloration of teeth and unpleasant taste [9]. So the use of herbal formulations as antiplaque agents is being explored, as these do not have any reported side effects enabling their use on a daily basis. Green tea is one such herbal preparation, which has potent antioxidant properties. When used as a mouth rinse, green tea reduced plaque formation and showed promise in preventing periodontal disease. Studies conducted over the last 20 years have shown that the green tea contains catechin compounds: that can inhibit the growth of a wide range of gram-positive and gram-negative bacterial species with moderate potency [10,11,12]. Catechin gallates such as ECg intercalate into phopsholipid bilayers and it is likely that they affect both virulence and antibiotic resistance by perturbing the function of key processes associated with the bacterial cytoplasmic membraneThus the present study was carried out as a prospective, randomized, placebo and positively controlled research designed to evaluate clinical effects of green tea in reduction of gingival inflammation in patients undergoing orthodontic treatment.

## Methodology:

After obtaining an informed consent, 40 healthy subjects undergoing orthodontic treatment who meet the inclusion criteria were randomly allocated to the study and control groups. All the subjects were examined using Loe and silness gingival index to obtain the baseline data. Examination was done using a mouth mirror, explorer and adequate illumination. An adequate number of instruments were supplied to avoid interruption in the examination, for need of sterilization of used instruments. After recording the index the subjects were randomly allocated in study group and in control group. 5 ml of mouthwash was given for 21 days, once daily under supervision to the subjects. Subjects were asked to rinse for one minute with the solution. The study group was given green tea mouthwash whereas control group was given placebo mouthwash without the active ingredient i.e. green tea. All the subjects were re-examined using Loe and silness gingival index after 21 days.

### Method of preparation of mouthwash:

Experimental solution: Fresh green tea was obtained from the market in the form of dip bags. 2% solution was prepared by brewing 2 grams of tea in 100 ml of water for 5 mins and then it was dispensed in sterile disposable containers.

Control solution: Similar procedure was applied to prepare the placebo mouthwash except the active ingredient i.e. green tea was not added. Respective mouthwashes were freshly prepared and dispensed to the subject's daily.

### Statistical analysis:

After 21 days the results obtained were recorded and statistically analyzed by (1) Mann Whitney U test and (2) Wilcoxon signed -rank test.

## Results and Discussion:

The scores before the use of mouthwash in study and control group were 1.39 ± .27 and 1.22 ± .14 respectively. The scores after 21 days in study and control group were 0.87 ± .25 and 1.22 ± .16 respectively. The study group and control group was statistically analyzed by Wilcoxon signed rank test in which the values before and after the use of mouthwash were compared. The p value was found to be statistically significant (p<0.05) in study group. But in control group statistical significant could not be reached.

A comparison between the groups was made by Mann Whitney U test before the use of mouthwash and the values of study group and control group were almost similar but the p value was found to be statistically significant. The comparison between the groups after the use of mouthwash was made and the p value was found to be statistically significant (p<0.05). Green tea is rich in antioxidants [13]. Antioxidants are substances that scavenge free radicals - damaging compounds in the body that alter cells, tamper with DNA (genetic material), and even cause cell death. Free radicals occur naturally in the body, but environmental toxins (including ultraviolet rays from the sun, radiation, cigarette smoke, and air pollution) also give rise to these damaging particles. Many scientists believe that free radicals contribute to the aging process as well as the development of a number of health problems, including cancer and heart disease. Antioxidants such as polyphenols in green tea can neutralize free radicals and may reduce or even help prevent some of the damage they cause. The healthy properties of green tea are largely attributed to polyphenols, chemicals with potent antioxidant properties. Polyphenols contained in teas are classified as catechins [10]. EGCG is the catechin that is found in maximum amount in the tea, it accounts for almost about 65% of catechin content in the tea. Green tea also contains alkaloids including caffeine, theobromine, and theophylline. These alkaloids provide green tea's stimulant effects. L-theanine, an amino acid compound found in green tea, has been studied for its calming effects on the nervous system. In recent years usage of herbal medicine has shown some good results, out of which usage of green tea as a mouth rinse has proved to be beneficial due to its antioxidant properties, which reduces gingivitis. In the course of our investigations of natural substances, which have the potential of effectively preventing gingivitis, we have focused on green tea as it is commonly consumed in the world especially in Asian countries. The catechins in green tea inhibit collagenase activity, the growth of S. mutans and the adherence of P. gingivalis to oral epithelial cells at concentrations below 0.25mg/ml. [12].

A total of 40 subjects undergoing orthodontic treatment were included in e this study. They were randomly divided into the study and control group. The age group chosen for study was 18-25 years, as these subjects are known to practice inadequate oral hygiene and alteration of body hormones, which makes them more susceptible to gingival and periodontal diseases. It was a double blind study, thus avoiding the investigator bias. Loe and Silness gingival index was used to measure the gingival status at the beginning and again after 21 days of using 5ml of mouthwash once daily. An improvement in the gingival score was noticed among all subjects in the study group at the end of 21 days; however no significant difference in the gingival score was seen in subjects using the placebo mouthwash. The improvement in the clinical parameters suggests that green tea catechins prevent the development of gingival disease. The study carried out by Lauten JD et al could not reach statistical significance in terms of differences between gingival index, plaque index or relative abundance of bacterial species for efficacy of the mouthrinse prepared from four plant species (Melaleuca alternifolia, Leptospermum scoparium, Calendula officinalis and Camellia sinensis), but this was attributed to the small study sample. Our study found a significant decrease of gingival scores by the continuous use of green tea as a mouthrinse and this result is in accordance with the studies carried out in the past by various authors [12, 13,14,15,13] which showed that green tea catechin has marked bactericidal effect against Porphyromonas gingivalis and Prevotella spp, reduces probing depth, can prevent gingivitis and further progress to periodontitis, thus acting not only as an antibacterial but also an anticariogenic material.

## Conclusion:

Green tea catechins potentially possess a significant effect on reducing plaque accumulation and gingivitis. There is a growing amount of in-vitro research identifying tea's potential oral health benefits. However, further longer term, well-controlled human trials are required to determine its long-term efficacy. In the mean time it is reasonable to conclude that the continuous use of green tea catechin as a mouth rinse on a daily basis will prove to be a useful, cost beneficial and practical method for early prevention of gingival diseases, which will be an important contribution to the dental public health.
